# Immune profiles and DNA methylation alterations related with non-muscle-invasive bladder cancer outcomes

**DOI:** 10.1186/s13148-022-01234-6

**Published:** 2022-01-21

**Authors:** Ji-Qing Chen, Lucas A. Salas, John K. Wiencke, Devin C. Koestler, Annette M. Molinaro, Angeline S. Andrew, John D. Seigne, Margaret R. Karagas, Karl T. Kelsey, Brock C. Christensen

**Affiliations:** 1 Department of Epidemiology, Geisel School of Medicine, Dartmouth College, Lebanon, NH 03766 USA; 2 Department of Neurological Surgery, University of California San Francisco, San Francisco, CA 94143 USA; 3 Department of Biostatistics and Data Science, University of Kansas Medical Center, Kansas City, KS 66160 USA; 4 Department of Neurology, Geisel School of Medicine, Dartmouth College, Lebanon, NH 03766 USA; 5 Department of Surgery, Section of Urology, Geisel School of Medicine, Dartmouth College, Lebanon, NH 03766 USA; 6 Departments of Epidemiology and Pathology and Laboratory Medicine, Brown University, Providence, RI 02912 USA; 7 Departments of Molecular and Systems Biology, and Community and Family Medicine, Geisel School of Medicine, Dartmouth College, Lebanon, NH 03766 USA; 8 Dartmouth Hitchcock Medical Center, 1 Medical Center Dr, 660 Williamson Translation Research Building, Lebanon, NH 03756 USA

**Keywords:** DNA methylation, Non-muscle-invasive bladder cancer, Immune profile, Immunomethylomic, Recurrence, Survival

## Abstract

**Background:**

Non-muscle-invasive bladder cancer (NMIBC) patients receive frequent monitoring because ≥ 70% will have recurrent disease. However, screening is invasive, expensive, and associated with significant morbidity making bladder cancer the most expensive cancer to treat per capita. There is an urgent need to expand the understanding of markers related to recurrence and survival outcomes of NMIBC.

**Methods and results:**

We used the Illumina HumanMethylationEPIC array to measure peripheral blood DNA methylation profiles of NMIBC patients (*N* = 603) enrolled in a population-based cohort study in New Hampshire and applied cell type deconvolution to estimate immune cell-type proportions. Using *Cox* proportional hazard models, we identified that increasing CD4T and CD8T cell proportions were associated with a statistically significant decreased hazard of tumor recurrence or death (CD4T: HR = 0.98, 95% CI = 0.97–1.00; CD8T: HR = 0.97, 95% CI = 0.95–1.00), whereas increasing monocyte proportion and methylation-derived neutrophil-to-lymphocyte ratio (mdNLR) were associated with the increased hazard of tumor recurrence or death (monocyte: HR = 1.04, 95% CI = 1.00–1.07; mdNLR: HR = 1.12, 95% CI = 1.04–1.20). Then, using an epigenome-wide association study (EWAS) approach adjusting for age, sex, smoking status, BCG treatment status, and immune cell profiles, we identified 2528 CpGs associated with the hazard of tumor recurrence or death (*P* < 0.005). Among these CpGs, the 1572 were associated with an increased hazard and were significantly enriched in open sea regions; the 956 remaining CpGs were associated with a decreased hazard and were significantly enriched in enhancer regions and DNase hypersensitive sites.

**Conclusions:**

Our results expand on the knowledge of immune profiles and methylation alteration associated with NMIBC outcomes and represent a first step toward the development of DNA methylation-based biomarkers of tumor recurrence.

**Supplementary Information:**

The online version contains supplementary material available at 10.1186/s13148-022-01234-6.

## Background

In 2021, the estimated number of bladder cancer deaths is projected to be 17,200, with an estimated number of new cases of 83,730 in the USA. Bladder cancer is the fourth most common cancer among men and twelfth most common among women, which may in part be due to smoking prevalence rates being higher in men than women as cigarette smoking accounts for half of all cases (47%) in the USA. [1]. Seventy-five percent of bladder cancers are diagnosed as low-grade non-muscle invasive tumors, NMIBC [2]. Cystoscopy is used for diagnosis (biopsy) with transurethral excision of localized tumors as the primary treatment [3–5]. Although transurethral excisions can successfully control the disease and mortality from bladder cancer among patients with localized tumors is low, 45% of NMIBC cases have recurrences within 12 months of surgery [6]. In addition, frequent invasive follow-up via cystoscopy without prognostic markers leads to significant patient morbidity and comes with a considerable cost burden to the health care system, estimated at approximately $4 billion dollars annually in the USA [7]. To control the patient and healthcare burdens associated with NMIBC, there is an imminent need for biomarkers to identify those at the highest risk of tumor recurrence.

Peripheral blood immune profiles have been associated with different outcomes in bladder cancer patients and may have clinical utility for NMIBC prognosis [8–13]. For instance, in NMIBC, patients with elevated neutrophil-to-lymphocyte ratio (NLR) had poorer cancer-specific survival than patients with lower NLR [9–11]. Thus, elevated NLR could be a potential predictor of overall survival and cancer-specific survival for this disease. Other studies have indicated that bladder cancer patients with increased lymphocyte-to-monocyte ratio had poorer overall survival and cancer-specific survival [12, 13]. In addition, Bacillus Calmette–Guérin (BCG), a commonly used intravesical immunotherapy for NMIBC administered post-surgery, has been reported to reduce the proportion of natural killer T cells, memory CD4T, CD8T, and regulatory T cells in the peripheral blood of NMIBC patients [8]. Together, circulating immune profiles might be promising markers for reducing adverse outcomes in NMIBC patients. Previous studies have relied on complete blood count differential (CBC) tests to determine immune profile variables [14, 15]. The CBC test relies on fresh blood samples and is incapable of providing proportions of specific lymphocyte subtypes [16].

Our prior work has established methods to infer the immune profiles in archival samples using immune cell-type-specific DNA methylation [17, 18]. DNA methylation plays an essential role in gene regulation for cell lineage specification [19, 20]. Differentially methylated regions (DMRs) have been used to distinguish cell types, including leukocyte subtypes, and form the basis of reference-based deconvolution methods for estimating specific immune cell-type proportions [18, 21, 22]. Compared with cytological methods for determining cell type abundances and proportions, such as flow cytometry, DNA methylation-based cell-type deconvolution does not require a fresh substrate, intact cells, or batch-sensitive reagents, is reproducible and cost-effective relative to time-sensitive blood processing [23, 24]. With cell-type deconvolution approaches, it is possible to identify immune cell-type profiles and test their relationship with cancer outcomes in archived samples [25]. This study used the archival blood samples of NMIBC patients to test the association between the immune profiles and outcomes in bladder cancer patients.

In the present study, we sought to identify immune profiles and epigenetic features associated with disease recurrence in the hope that such information might help improve the management of NMIBC. Here, we hypothesized that CpG-specific DNA methylation and DNA methylation-derived immune cell profiles are associated with recurrence-free survival in NMIBC patients. We used archival blood samples from a population-based case–control study to obtain genome-scale DNA methylation profiles. We then investigated the association between the methylation-derived immune profiles and outcomes in NMIBC patients. Preliminary work from our group observed an association between methylation-derived NLR (mdNLR) and survival in bladder cancer patients using a smaller sample size (223 cases) and an early genome-scale methylation array (HumanMethylation27K array) [26]. In this study, we increased the sample size (603 cases), used a new more comprehensive array (HumanMethylationEPIC array) with 30 times as many measured features, and a new cell type deconvolution library [18] to estimate immune cell-type proportions. An epigenome-wide association study (EWAS) and enrichment analyses were used to determine possible CpG sites and gene sets associated with recurrence-free survival.

## Results

**Fig. 1:**
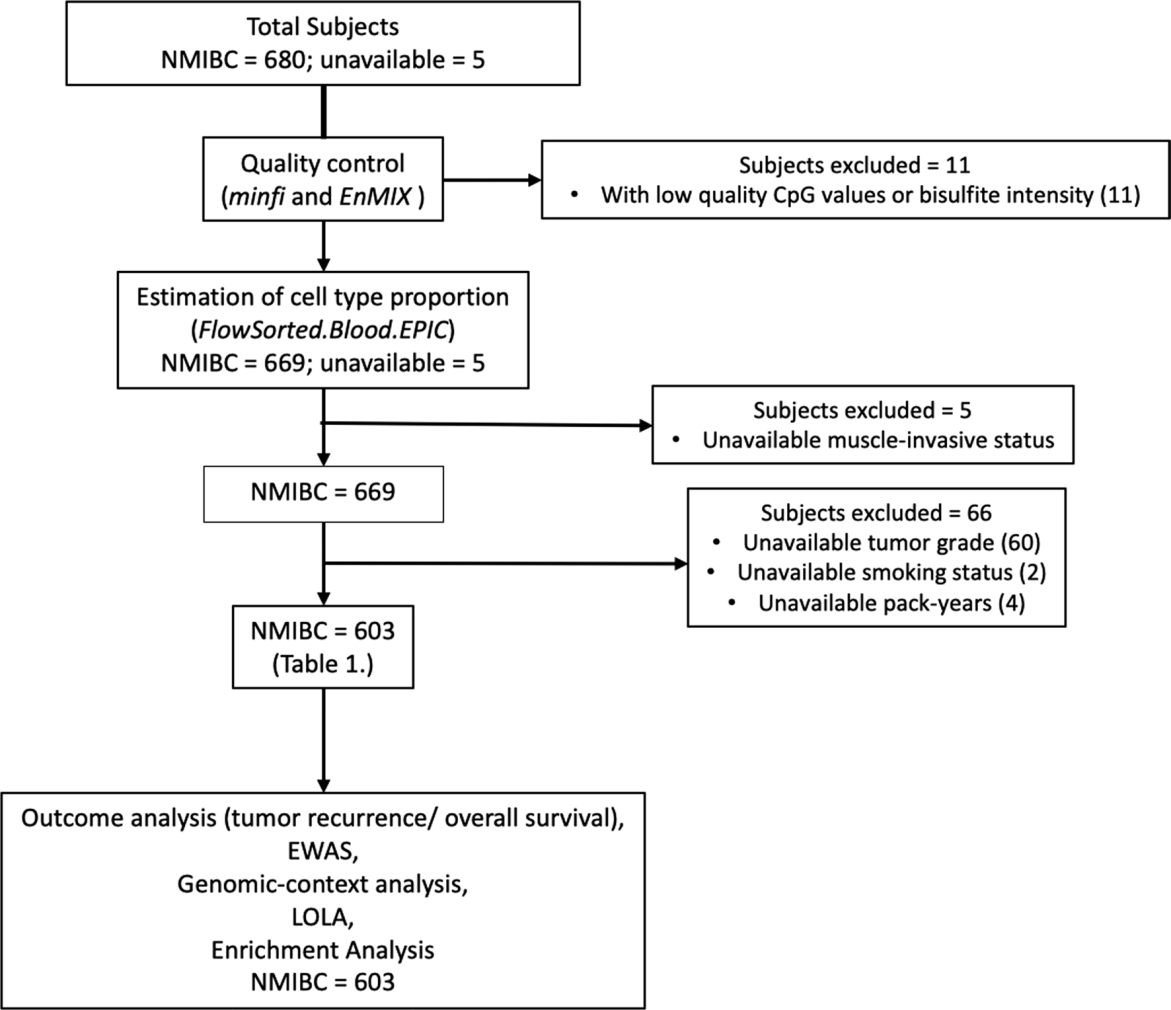
Flow chart of study

Profiles of DNA methylation were obtained from 685 peripheral blood samples using the Human MethylationEPIC array. Eighty-two subjects were excluded due to low-quality CpG value or bisulfite intensity (*n* = 11), or without muscle-invasive status, histopathology re-review, tumor grade, smoking status, and pack-years (*n* = 71) (Fig. 1). The remaining subjects (*N* = 603) included in the study group were 75.8% men (*n* = 457), 82.9% ever-smokers (*n* = 500), and had a median age of 66 (Table 1). We estimated the cell-type proportions for each patient using methylation values by performing *FlowSorted.Blood.EPIC* (see Additional file 7: Figure S1 for the distribution). Neutrophil-to-lymphocyte ratio (NLR) was then calculated according to the ratio of neutrophil proportion to lymphocyte proportion (B cell + CD4T cell + CD8T cell + NK cell), and the median methylation-derived NLR (mdNLR) was 1.97. Further details of study population characteristics are described in Table 1.

**Table 1:** Characteristics of subjects after excluding subjects with missing values

	NMIBC (*n* = 603)
*Age*	
Median (Q1, Q3)	66 (57,71)
*Sex*	
Male	457 (75.8%)
Female	146 (24.2%)
*Tumor grade*	
Grade 1	290 (48.1%)
Grade 2	162 (26.9%)
Grade 3	125 (20.7%)
Grade 4	26 (4.3%)
*Smoking status*	
Never	103 (17.1%)
Ever	500 (82.9%)
*BCG: immunotherapy*	
No	514 (85.2%)
Yes	89 (14.8%)
*NLR*	
Median (Q1, Q3)	1.97 (1.40, 2.93)
*10-year dead status*	
Alive	423 (70.1%)
Deceased	180 (29.9%)
*10-year survival*	
Median (Q1, Q3)	120.0 (105, 120)
*10-year recurrence status*	
No	193 (32.0%)
Yes	295 (48.9%)
Missing	115 (19.1%)
*10-year recurrence*	
Median (Q1, Q3)	17.7 (6.9, 67.1)
Missing	115

Exclude subjects with missing values in muscle-invasive status, pathology reviewing status, tumor grade, smoking status, or pack-years

**Fig. 2:**
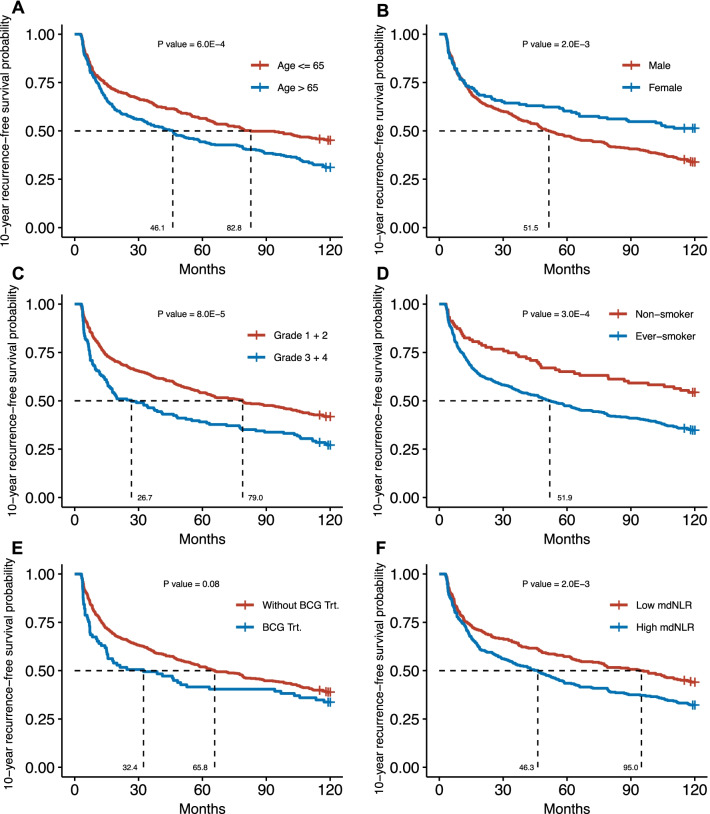
Kaplan–Meier analysis of 10-year recurrence-free survival (RFS). 10-year RFS curves stratified by **A** age, **B** sex, **C** tumor grade, **D** smoking status, **E** BCG treatment status or **F** mdNLR level. *P*-values for log-rank tests are shown

To characterize 10-year recurrence-free survival (RFS), we generated Kaplan–Meier curves for each covariate and fit a Cox proportional hazard regression model for univariate analyses and multivariable analyses, respectively. In the Kaplan–Meier analysis, the NMIBC patients aged > 65 had a worse probability of RFS compared with the NMIBC patients with age ≤ 65 (*P* = 0.0006). Females had a greater probability of RFS compared with males (*P* = 0.002), and the NMIBC patients with high-grade tumors (grade 3 + 4) had a lesser probability of RFS than those with low-grade tumors (grade 1 + 2) (*P* = 8.0 × 10^−5^). Ever-smokers had a worse probability of RFS than the NMIBC patients who were never smokers (*P* = 3.0 × 10^−4^). NMIBC patients with low mdNLR had a greater probability of RFS than the patients with high mdNLR (*P* = 0.002) (Fig. 2). Consistent with the Kaplan–Meier results, in a multivariable Cox model, age > 65 (HR = 1.01, 95% CI = 1.00–1.03), high tumor grade (HR = 1.48, 95% CI = 1.17–1.87), ever-smoking (HR = 1.65, 95% CI = 1.22–2.25), and mdNLR (HR = 1.12, 95% CI = 1.04–1.20) were significantly associated with an increased hazard of RFS (Table 2).

**Table 2:** Cox proportional hazards 10-year recurrence-free survival models

	*n* (%) event^#^	*n* (%) no-events^#^	Event occurrence months		Multivariable^$^ model	
			Mean	Median	HR (95% CI)	*P* value
Age	373 (61.9)	230 (38.1)	65.6	62.0	1.01 (1.00–1.03)	0.024
*Tumor grade*						
1 + 2	263 (58.2)	189 (41.8)	69.9	79.0	Referent group	
3 + 4	110 (72.8)	41 (27.2)	52.8	26.7	1.48 (1.17–1.87)	1.0E−3
*Smoking status*						
Non-smoker	47 (45.6)	56 (54.4)	82.3	120.0	Referent group	
Ever-smoker	326 (65.2)	174 (34.8)	62.2	51.9	1.65 (1.22–2.25)	1.0E−3
mdNLR*	373 (61.9)	230 (38.1)	65.6	62.0	1.12 (1.04–1.20)	3.0E−3

HR: hazard ratio, CI: confidence interval, mdNLR: methylation-derived neutrophil to lymphocyte ratio

Stratification was used on sex and BCG treatment status for proportional assumption

^$^The model controlling for age, sex, tumor grade, smoking status, BCG treatment status, and mdNLR

^*^Winsorization was used on the top 2% values for fitting linearity assumption

^#^Initial recurrence or the death whose cause was unknown

We next investigated the association between immune cell-type proportions and RFS in multivariable models. CD4T (HR = 0.98, 95% CI = 0.97–1.00) and CD8T cell proportion (HR = 0.97, 95% CI = 0.95–1.00) were significantly associated with the decreased hazard of RFS. Monocyte cell proportion (HR = 1.04, 95% CI = 1.00–1.07) was significantly associated with the increased hazard of RFS. Both B cell and NK cell proportion hazard estimates were < 1 but not statistically significant (Additional file 1). We also examined the 10-year overall survival (OS) in univariate models and the multivariable models. Observed associations with RFS were consistent for OS for: age, male, high tumor grade, ever-smoking, and mdNLR (Table 3 and Additional file 7: Figure S2). In addition, CD4T, CD8T, B cell, and NK cell proportion were significantly associated with the decreased hazard of death; neutrophil cell proportion was significantly associated with the increased hazard of death (Additional file 2).

**Table 3:** Cox proportional hazards 10-year overall survival models

	*n* (%) deceased	*n* (%) alive	Survival months	Multivariable model	
			Mean^#^	HR (95% CI)	*P* value
Age	180 (29.9)	423 (70.1)	104.3	1.07 (1.04–1.08)	8.7E−9
*Sex*					
Male	156 (34.1)	301 (65.9)	102.2	Referent group	
Female	24 (16.4)	122 (83.6)	111.1	0.62 (0.40–0.96)	0.032
*Tumor grade*					
1 + 2	118 (26.1)	334 (73.9)	106.6	Referent group	
3 + 4	62 (41.1)	89 (58.9)	97.7	1.54 (1.12–2.12)	8.0E−3
*Smoking status*					
Non-smoker	19 (18.4)	84 (81.6)	110.8	Referent group	
Ever-smoker	161 (32.2)	339 (67.8)	103.0	1.66 (1.03–2.67)	0.039
*BCG treatment*					
No	152 (29.6)	362 (70.4)	104.8	Referent group	
Yes	28 (31.5)	61 (68.5)	101.9	1.04 (0.68–1.60)	0.842
mdNLR*	180 (29.9)	423 (70.1)	104.3	1.46 (1.32–1.60)	6.0E−15

HR: hazard ratio, CI: confidence interval, mdNLR: methylation-derived neutrophil to lymphocyte ratio

^*^Winsorization was used on the top 2% values for fitting linearity assumption

All covariates modeled met proportionality assumptions ^#^: the median survival month for each variable is 120

**Fig. 3:**
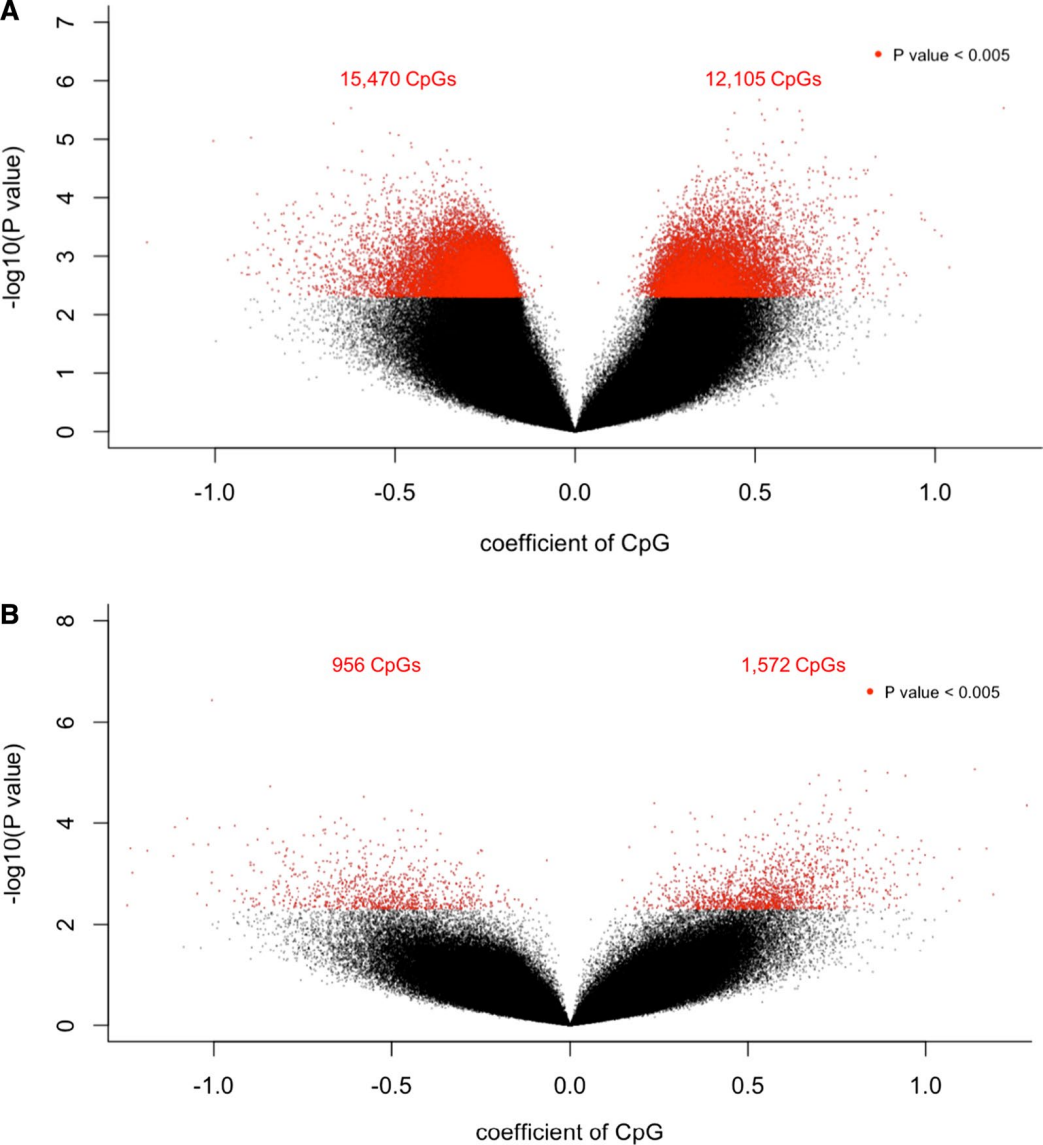
Volcano plots of recurrence-free survival (RFS) associated CpGs from the epigenome-wide association study (EWAS) analyses. The Cox multivariable model that was fitted in EWAS was shown in each plot. CpGs are colored in red (**A**) 12,105 CpGs were associated with the increased hazard of NMIBC 10-year RFS, and 15,470 CpGs were associated with the decreased hazard of NMIBC 10-year RFS. (**B**) 1572 CpGs were associated with the increased hazard of NMIBC 10-year RFS, and 956 CpGs were associated with the decreased hazard of NMIBC 10-year RFS

Next, we assessed the relation of NMIBC patient outcomes with DNA methylation. First, Cox proportional hazards models were fit for each CpG controlling for age, stratified sex, tumor grade, smoking status, and stratified BCG receiving status. Without adjustment for immune cell proportions, we identified 27,575 CpGs whose methylation was associated with a significant (*P* < 0.005) difference in hazard of RFS (Fig. 3A, Additional file 3). We then conducted similar analyses controlling for age, sex, tumor grade, smoking status, BCG status, and winsorized immune cell proportions. As expected and demonstrating the importance of adjusting for cell-type proportions, the fully adjusted models were attenuated and identified 2528 CpGs whose methylation was associated with a significant difference (*P* < 0.005) in the hazard of RFS. The 10 CpGs most strongly (with smallest *P*-value) associated with hazard of RFS corresponded to 10 genes: *TMCO4* (cg04738197), *LENG9* (cg12057190), *CDC42EP5* (cg12057190), *LNP1* (cg02540094), *TOMM70A* (cg02540094), *RUNX2* (cg08012149), *TBXAS1* (cg01584377), *SSH1* (cg16237760), *SFXN2* (cg08609163), and *COG3* (cg06172950). 1572 CpGs were associated with the increased hazard of RFS, and 956 CpGs were associated with the decreased hazard of RFS (Fig. 3B). The complete list of RFS-associated CpGs is provided in Additional files 4 and 5.

**Fig. 4:**
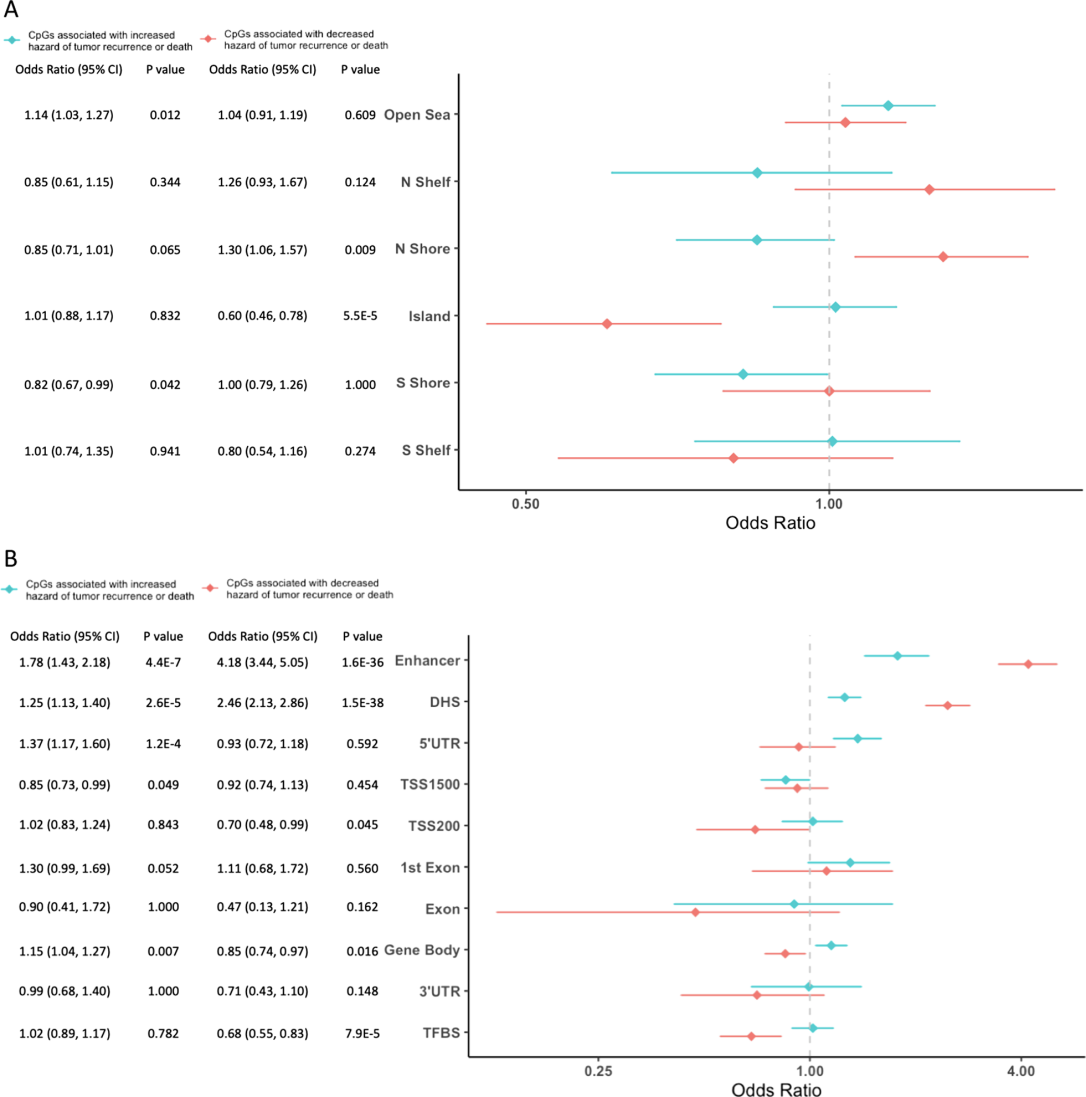
Genomic context enrichment analysis of CpG sites whose methylation state is significantly associated with recurrence-free survival. Enrichment analysis of (**A**) relation to CpG island and (**B**) genomic context of NMIBC recurrence-free survival associated CpGs. The 2528 CpGs from EWAS (*P* < 0.005) were tested for enrichment versus all modeled CpGs. The bar represents the 95% confidence interval. Mantel–Haenszel was used to test RFS-associated CpGs enrichment of CpG island-related gnome context. An odds ratio larger than 1 means enrichment, and an odds ratio smaller than 1 indicates depletion

To gain a better understanding of the regions where the hazard-associated CpGs were located, we tested for enrichment of CpG island region context among CpG loci associated with a significant change in the hazard of RFS. We found that 1572 CpGs associated with the increased hazard of RFS were significantly enriched in the open sea (OR = 1.14, 95% CI = 1.03–1.27) and were significantly depleted in CpG island S Shore regions (OR = 0.82, 95% CI = 0.67–0.99). The 956 CpGs associated with a decreased hazard of RFS were significantly enriched in CpG island N Shore regions (OR = 1.30, 95% CI = 1.06–1.57) and were significantly depleted in CpG island (OR = 0.60, 95% CI = 0.46–0.78) (Fig. 4A). We also tested for enrichment of other gene regulatory regions among CpG loci associated with a significant change in the hazard of RFS.

Location of CpGs in regulatory regions also was investigated and the CpGs associated with the increased hazard of RFS were significantly enriched in enhancer regions (OR = 1.78, 95% CI = 1.43–2.18), DNase hypersensitive sites (DHS) (OR = 1.25, 95% CI = 1.13–1.40), 5’UTR regions (OR = 1.37, 95% CI = 1.17–1.60) and gene body (OR = 1.15, 95% CI = 1.04–1.27); however, these CpGs were significantly depleted in regions 200–1500 bps upstream of the transcription start site (TSS1500) (OR = 0.85, 95% CI = 0.73–0.99). In addition, while the CpGs associated with a decreased hazard were strongly enriched in enhancer regions (OR = 4.18, 95% CI = 3.44–5.05) and DHS (OR = 2.46, 95% CI = 2.13–2.86), they were depleted for TSS200 (OR = 0.70, 95% CI = 0.48–0.99), gene body (OR = 0.85, 95% CI = 0.74–0.97) and transcription factor binding sites (TFBS) (OR = 0.68, 95% CI = 0.55–0.83) (Fig. 4B). Using the CpGs associated with hazard of RFS from cell-type unadjusted models in tests for enrichment gave results that were largely consistent with those above based on CpGs from fully adjusted models (Additional file 7: Figure S3).

To further understand the biological function of the hazard-associated CpGs, Gene Set Enrichment Analysis (GSEA) Molecular Signature Database (MSigDB) was used to explore the potential gene sets which might associate with the tumor recurrence or death of NMIBC patients. The input was 2528 RFS associated CpGs from the Cox model EWAS adjusting for immune cell composition. In the top 10 hazard-associated gene sets in gene ontology (GO) terms, some gene sets were related to neurological system processing (Additional file 7: Figure S4A), and most related genes were associated with the increased hazard of RFS in NMIBC (Additional file 7: Figure S4B). Since mdNLR was associated with the hazard of recurrence or death of NMIBC, we were interested in immune-related gene sets. We checked the immunologic signature gene set, and the top 10 gene sets and their genes were related to immune cell regulation. However, only one gene set (BCELL VS MDC UP: up-regulated genes in B cells compared with myeloid dendritic cells after vaccination for influenza) consisting of 41 genes was significantly associated with RFS (FDR < 0.05) (Additional file 7: Figure S4C-D). Results from GO term analyses using 27,575 CpGs from the cell-type unadjusted models identified 5 pathways related to immunologic regulation among the top 10 pathways. Further, in the immunologic signature gene set specifically, we observed the top 10 pathways were associated with monocytes and lymphocytes (Additional file 7: Figure S5).

Locus overlap analysis (*LOLA*) was used to test the enrichment of CpGs in genomic regions. As our analysis was conducted on blood samples, results focus on the genomic regions within hematopoietic stem cells. When controlling for immune cell profiles, the 2528 CpGs associated with the hazard of RFS in NMIBC patients were most significantly enriched in Histone H3 acetylated at lysine 9 and 14 (H3K9K14ac) (*Q*-value = 1.9 × 10^−20^) (Additional file 7: Figure S6A). *LOLA* results for the 27,575 CpGs associated with the hazard of RFS from cell type unadjusted models were most significantly enriched in cistrome of the promyelocytic leukemia protein (PML) (*Q*-value < 0.05) (Additional file 7: Figure S6B).

We also ran a differentially methylated regions analysis using *DMRcate*. In this study, we found 11 CpGs in a specific genome region overlapping with the gene *BLCAP*. NMIBC patients without tumor recurrence or death within 10 years had a higher mean of methylation levels for this region compared with NMIBC patients with tumor recurrence or death within 10 years, and one CpG in this region was significant (FDR = 2.63 × 10^−14^) (Additional files 6, 7: Figure S7).

## Discussion

In this study, we tested whether immune profiles and epigenetic features are associated with NMIBC recurrence. Although previous work observed the association between NLR and overall survival in NMIBC patients, it was in a smaller study sample and used DNA methylation data from a dated (second generation) array platform with ~ 27,000 CpGs. In this study, our sample size was nearly three times larger and DNA methylation data was collected using the current genome-scale platform (fourth generation), measuring ~ 860,000 CpGs for which an optimized cell-type deconvolution library exists to determine highly accurate immune cell-type proportions. We extended tests of association with the patient outcomes beyond the methylation-derived neutrophil-to-lymphocyte ratio (mdNLR) to include leukocyte-specific cell-type proportions. Our findings suggest that elevated mdNLR increased the hazard of RFS in NMIBC patients. These findings are consistent with previous studies demonstrating that NLR was significantly higher in high-risk NMIBC patients [27], and increased NLR was positively associated with poor prognosis [28, 29]. While past studies have shown a significant association between NLR and outcomes in NMIBC patients using the conventional method of CBC tests [30–32], this study uses NLR derived from blood methylation profiles. Compared with flow cytometry, estimation using the differentially methylated regions (DMR) library has several advantages: it does not require long sample processing time, large volume of blood, or intact cells as DNA and 5-methylcytosine are both stable [33]. The advantage of our approach is the ability to use archived samples that many investigators may already have. Moreover, utilizing blood samples to monitor patient outcomes is less invasive compared with a cystoscopy, the routine screening method. For NMIBC patients with a high risk of tumor recurrence, BCG is the standard intravesical immunotherapy to induce immune system eliminating bladder cancer cells that might be left after surgery [8, 34, 35], and therefore, blood immune profile is a potential prognostic factor. Immune profiling with DNA methylation data is a promising avenue for assessing NMIBC prognosis.

As NLR is composed of lymphocyte and neutrophil proportions, we also considered the association between each methylation-derived immune cell type proportion and patient outcomes. Interestingly, increased CD4T and CD8T cell proportions were associated with decreased NMIBC recurrence-free survival and overall survival. Prior work has shown that NMIBC patients with a high CD4T cell count in BCG pretreatment microenvironment have a significantly prolonged recurrence-free survival compared to patients with a low CD4T cell count [35]. In addition to the immune cell types explored in this study, other cell types have been shown to affect NMIBC development, such as GATA3 + T cells, regulatory T cells, and tumor-associated macrophages [35]. Other work showed an increased CD8^+^ILT2^+^ T cell proportion was associated with a significantly increased hazard of NMIBC recurrence [36]. Further, peripheral neutrophil x platelet/lymphocyte was inversely correlated with high-risk NMIBC recurrence-free survival [37]. These results demonstrate the potential of immune cell profiles in evaluating the prognosis of NMIBC patients, and future work mapping DNA methylation profiles of additional immune cell types and states could add detail to investigations of immune profiles and bladder cancer outcomes. In addition, peripheral immune cell distribution is affected by potentially residual confounding factors such as, infection [38], inflammation [39], lifestyles, treatments [40, 41], obesity [42], chronic alcohol consumption [43], and type 2 diabetes [44]. Since our study includes hundreds of subjects and has a decade or more of follow-up time, in the future, we will re-explore the effect of these residual confounding factors on the association of circulating immune cell distribution and NMIBC outcomes. In the future, we will perform higher resolution methylation cell mixture deconvolution to resolve additional immune cell types and employ blood count on prospectively collected samples. Together, these data will allow us to understand if specific subsets of neutrophils or lymphocytes contribute to high NLR in patients with poor outcomes.

Bacillus Calmette–Guérin (BCG) is the most commonly used immunotherapy for high-risk NMIBC. After transurethral resection of bladder tumors, NMIBC patients may receive BCG intravesical therapy to induce an immune response in the bladder to attack cancer cells. Previous studies have shown that tumor immune environments may interact with BCG and interfere with the efficacy of this therapy. For instance, IL-12 secreted by BCG-induced monocytes was increased in NMIBC patients without tumor recurrence, a phenomenon that may involve the innate immune memory of circulating monocytes [45]. Nevertheless, patients receiving BCG in our study did not have peripheral immune cell profiles that differed from patients who were BCG naïve, and BCG was not significantly associated with NMIBC outcomes. As the number of patients with BCG treatment was relatively low (*N* = 89; 14.8%) and limited power to observe possible therapy induced changes in peripheral immune profiles, future work is needed to address the potential associations of BCG with peripheral immune profiles and patient outcomes. Most BCG-treated patients were already high-risk. In addition, blood samples were collected only after surgery or initial BCG treatment. To assess the changes in immune profiles over time, having multiple blood draws is necessary for future work.

This EWAS identified several CpGs associated with NMIBC recurrence-free survival when controlling for stratified sex, age, tumor grade, smoking status, stratified BCG receiving status, and immune cell profiles. With a *P*-value < 0.005, 2528 CpGs were found to be associated with the hazard of RFS.

Among the top CpGs whose methylation was associated with NMIBC recurrence or death in the fully adjusted models, some genes have been previously associated with bladder cancer. Slingshot homolog-1 (*SSH1*) had a positive association with tumor grade, tumor invasion, and tumor recurrence of bladder cancer patients [46]. Runt-related transcription factor 2 (*RUNX2*) is a key factor of osteoblast differentiation and has been reported to be associated with epithelial-mesenchymal transition in bladder tumors. Furthermore, *RUNX2* could predict early recurrence in bladder cancer patients with high accuracy [47, 48]. Similar to past studies, *SSH1* and *RUNX2* were associated with the increased risk of tumor recurrence in our study. In addition, NMIBC patients with tumor recurrence within ten years had significantly higher methylation levels in the gene body of these two genes compared with patients without tumor recurrence.

When not adjusting for immune cell profiles, 27,575 CpGs were associated with the decreased hazard of RFS. The top 10 CpGs with the most significant *P*-value corresponded to 6 genes: *BCL11A* (cg24361098), *TMCO4* (cg04738197), *MICALCL* (cg01518090), *GRAP2* (cg21012238), *TRAM2* (cg15085626), and *KIRREL* (cg10570484). While these genes have not been reported to be associated with bladder cancer outcomes, they have been reported to be involved in mechanisms promoting cancer development in tumors [49–52] or blood [53, 54]. Although we measured blood methylation in this study, we plan to measure tumor methylation and will explore the association of CpGs in these genes with NMIBC outcomes. What was more interesting is that the model, with or without controlling for immune cell profiles, led to different results. While immune cell profiles are usually not adjusted in the Cox model, we controlled for immune cell profiles since the immune system plays a key role in tumor development. This study presents a new perspective to demonstrate the difference between models with or without adjusting for immune profiles, indicating the need for further investigation on the involvement of immune profiles in outcome analyses.

Through DMR analysis, we found NMIBC patients with tumor recurrence or death within 10 years had a lower methylation level in the *BLCAP* region compared with patients without tumor recurrence or death. The bladder cancer-associated protein (*BLCAP*) gene encodes a protein that stimulates apoptosis. It has been reported that loss of protein expression is associated with bladder tumor progression, and the application of staining patterns for this protein could be a potential biomarker in bladder cancer [55]. In functional analysis, strong nuclear expression of *BLCAP* was associated with expression of p-*STAT3* and overall poor disease outcome. Additionally, *BLCAP* was discovered to interact with *STAT3* physically and may involve the *STAT3*-mediated progression of precancerous lesions to invasive bladder tumors [56]. These results were consistent with our finding that NMIBC patients with poor outcomes had lower methylation levels in the *BLCAP* region. Since the model we used for DMRs analysis was adjusted for immune cell profiles, we are curious whether immune cells may play roles in the interaction between *BLCAP* and *STAT3* and will investigate this in the future. The CpG site with significantly lower methylation (cg10642330) is located in the 5’ UTR of *BLCAP* and the gene body of *NNAT*. The methylation beta value of this CpG site in *NNAT* had been reported significantly higher in prostate cancer tissue relative to adjacent normal tissues [57]. Though no CpG site in the *BLCAP* region was significantly associated with the hazard of tumor recurrence or death in the adjusted EWAS, five *BLCAP* CpGs (cg26083330, cg23757721, cg13790727, cg03061677, and cg04489586) were associated in the model not controlling for immune cell proportions (not including the DMR analysis site cg10642330). In the future, we will investigate the relation of *BLCAP* tumor methylation with survival outcomes.

Our results reveal several features of peripheral blood immune profiles that are associated with outcomes in NMIBC patients. We showed that higher CD4 or CD8 proportions were associated with decreased hazard of recurrence or death and further established that high NLR is associated with an increased hazard of RFS. The EWAS portion of our study also points to epigenetic reprogramming within the immune compartment being involved in tumor recurrence of NMIBC patients. Future study in a prospective setting will assess the clinical utility of incorporating methylation in predicting hazard of recurrence and shaping recommendations for disease surveillance. In addition to immune cells, future work examining cell-type proportions in tumor microenvironments of NMIBC patients is needed to understand the relationship between peripheral immune profiles with tumor-infiltrating immune profiles and patient outcomes. This work contributes to our understanding of associations of methylation-derived immune profiles and NMIBC patient outcomes and could further contribute to developments in epigenetic biomarkers of cancer.

## Conclusions

Here we demonstrate the associations of non-muscle-invasive bladder cancer outcomes with immune profiles. In addition, we identify preliminary evidence of discrete and regional CpG methylation associations with bladder cancer outcomes. The findings could contribute to developing epigenetic biomarkers for recurrence-free survival in non-muscle-invasive bladder cancer.

## Methods

### Study subjects and samples

The subjects and data used in this study are described in more detail in prior publications [58–60]. Briefly, subjects were recruited from all three phases of a New Hampshire population-based bladder cancer case–control study [61]. The first wave of this study (phase 1) collected blood samples from 331 individuals diagnosed with incident bladder cancer between July 1994 and June 1998. The second study wave (phase 2) collected blood samples from 243 individuals diagnosed between July 1998 and December 2001. Finally, the third study wave (phase 3) obtained blood samples from 194 individuals recruited and diagnosed between July 2002 and December 2004. All the subjects were identified using the New Hampshire State Cancer Registry, hospital pathology departments, and hospital cancer registries, and all blood samples were collected after the time of diagnosis (time range: 20–1790 days). Among patients, 40 patients received BCG treatment in phase 1, 29 patients received BCG in phase 2, and 19 patients received BCG in phase 3. All patients with BCG treatment had blood drawn after receiving BCG (time range: 7–1542 days). An outline of data filtering and inclusion/exclusion criteria applied to these data are shown in Fig. 1. Briefly, subjects without muscle-invasive status, histopathology re-review, tumor grade, smoking status, or pack-years were removed from the study. Subjects that withstood the aforementioned exclusion criteria were retained and used in downstream statistical analyses.

### DNA extraction, quantification, and bisulfite modification

Each blood sample was maintained at 4C and frozen within 24 h of blood draw. A hundred μl buffy coat was used to extract DNA. The QIAMP DNA blood & Tissue kit was used to extract DNA from blood samples according to the manufacturer’s protocol. Extracted DNA was quantified by using the Qubit 3.0 Fluorometer. After bisulfite modification, an established 5 mC microarray protocol optimized for Illumina methylation arrays was used to determine the genome-wide 5mC profile. DNA samples were subjected to bisulfite conversion (according to the manufacturer’s protocol of the Zymo EZ DNA methylation Kit) with an input of 750 ng per sample and whole-genome amplified prior to array hybridization. Recovered substrate ssDNA were submitted for DNA methylation array processing.

### DNA methylation data

Bisulfite-modified DNA samples were measured for their DNA methylation status using the MethylationEPIC array, which interrogates > 860,000 CpG sites. Probe intensity data (iDAT files) from the EPIC methylation array were processed for quality control via the R package *minfi* [62] and *ENmix* [63] in R version 3.6. After quality control, 11 samples with low-quality CpG values or bisulfite intensity (threshold: 7000) were excluded from the study. The data were then normalized and conducted background correction through preprocessNoob procedure from *minfi*. The *ComBat* [64] was used to adjust for potential batch effect. Probes with a detection *P* > 1.0 × 10^−6^ in more than 10% of the samples were excluded (32,414). Also, 98,826 probes, which are cross-reactive, SNP-associated, and non-CpG (CpH) methylation [65], as well as 17,120 probes on sex chromosomes, were excluded. In total, 726,856 probes were used in downstream statistical analyses in downstream statistical analyses. *IlluminaHumanMethylationEPICanno.ilm10b4.hg19* [66] was used to annotate CpG sites. Relation to CpG island was defined by the “*Relation_to_Island*” as in the Illumina annotation used in the genomic context analysis. ‘5’UTR,’ ‘Exon,’ ‘Gene Body,’ and ‘3’UTR’ contexts were defined by having ‘5UTR,’ ‘ExonBnd,’ ‘Body’ and ‘3UTR’ in *UCSC_RefGene_Group*. ‘Enhancer’ context was defined by having a record in the *Phantom5_Enhancers*. ‘DHS’ context was defined by finding a record in the *DNase_Hypersensitivity_NAME*. ‘TFBS’ context was defined by having a record in the *TFBS_NAME*.

### Statistical analysis

The estimation of cell-type proportions was processed through the *estimateCellCounts2* from the *FlowSorted.Blood.EPIC* package in Bioconductor (version 3.9; [18]). Methylation-derived neutrophil-to-lymphocyte ratio (mdNLR) was calculated by performing cell-mixture deconvolution to estimate the proportion of leukocyte subtypes and the ratio of neutrophil proportion to lymphocyte proportion was then computed. Individual leukocyte cell-type proportions and mdNLR were included in outcome analyses as continuous variables for Cox proportional hazard regression. In addition, mdNLR was dichotomized based on the median mdNLR for the Kaplan–Meier method.

Ten-year overall survival was defined as the time interval from the date of initial diagnosis to death within 10 years (all deaths were related with bladder cancer). Subjects who were alive or lost to follow-up were censored at the last follow-up. Ten-year recurrence-free survival was defined as the time interval from the date of initial diagnosis to the first tumor recurrence or death (all causes), whichever occurred first within 10 years. Patients alive and free of the disease or lost to follow-up were censored at the last follow-up. For both overall and recurrence-free survival, patient survival times over 10 years were truncated at 10 years, and patients were censored if the first tumor recurrence or death occurred after 10 years. The median survival times for the two survival outcomes were estimated using the Kaplan–Meier method. In multivariable analyses, Cox proportional hazard regression models were used to examine the association of each variable on bladder cancer outcomes and were fit via *coxph* in the *survival* R package. The proportional hazards assumption was tested by using *cox.zph* from the *survival* R package. The *cox.zph* function tests the proportionality of all the predictors in Cox models by creating interactions with time. As sex and BCG treatment status violated the proportional hazards assumption, stratification on both variables was included. The linearity assumption was examined via *ggcoxfunctional* from the R *survminer* package, and methylation-derived immune cell profiles were found to violate the linearity assumption. Hence, winsorization was used on methylation-derived immune cell profiles. The winsorization cut-point of each immune cell profile is shown in Additional file 7: Figure S1. A *P*-value of < 0.05 was the significance threshold on multivariable analysis. Cox model results were presented using the *stargazer* R package.

### Epigenome-wide association study (EWAS), enrichment analysis, and differentially methylated regions (DMRs)

An epigenome-wide association study (EWAS) was performed using ewaff R package (https://github.com/perishky/ewaff) to investigate the association of CpG-specific DNA methylation and bladder cancer recurrence. We fit Cox proportional hazards models independently to each CpG, controlling for age, sex, tumor grade, smoking status, Bacillus Calmette–Guérin (BCG) receiving status, and immune cell profiles. Since all *P*-values adjusted for false discovery rate from EWAS results are higher than 0.05, we relaxed the threshold for genomic context and enrichment analyses using a *P*-value of < 0.005.

For CpG sites associated (*P* < 0.005) with bladder tumor recurrence, we examined whether those CpGs were enriched in CpG island-related genomic context or regulatory regions via using Mantel–Haenszel tests, adjusted for Illumina probe type to eliminate the difference in distributions on genomic context. CpG island-related genomic context includes open sea, north shelves, north shores, islands, south shores, and south shelves. Regulatory regions include enhancers, DNase hypersensitivity sites, 5’UTR, TSS1500, TSS200, 1st Exon, Exon, gene body, 3’UTR, and transcription factor binding sites. Next, the Locus Overlap analysis (*LOLA*) R package in Bioconductor (version 1.20.0; [67]) was used to investigate enrichment of genomic regions limited to tissue equal to “hematopoietic stem cell.” Finally, *gometh* and *gsameth* from the *missMethyl* package in Bioconductor (version 1.6.2; [68]) were used to test the enrichment of gene sets for Gene Ontology (GO) terms and the C7: immunologic signature gene set in the Gene Set Enrichment Analysis (GSEA) Molecular Signature Database (MSigDB) *cnetplot* from the *enrichplot* package in Bioconductor (version 1.10.1; https://yulab-smu.top/biomedical-knowledge-mining-book/) was used to plot gene-concept network plots.

Differentially methylated regions were identified and extracted through the *dmrcate* and *extractRanges* from the *DMRcate* R package [69]. The inputs were logit-transform of beta values (M-values). The phenotype of interest for comparison was “NMIBC patients with tumor recurrence within ten years or not” in our designed model. In addition, the designed model was adjusted for sex, age, tumor grade, smoking status, BCG receiving status, and immune cell profiles. We relaxed the threshold for DMRs analysis using an FDR-corrected *P*-value of < 0.1. Then, the *visualizeGene* from the *sesame* package [70] was used for observing the methylation levels of CpGs in regions identified by *DMRcate*.

## Supplementary Information

**Additional file 1**: Cox proportional hazards 10-year recurrence-free survival. Created via using *stargazer()* function

**Additional file 2**: Cox proportional hazards 10-year overall survival. Created via using *stargazer()* function

**Additional file 3**: The 27,575 CpGs associated with the hazard of 10-year RFS when the model without adjusting for immune cell profiles. The list of the 27,575 CpGs which were associated with the hazard of 10-year RFS from the EWAS with Cox model without adjusting for immune cell profiles.

**Additional file 4**: The 1,572 CpGs associated with the increased hazard of 10-year RFS. The list of the 1,572 CpGs which were associated with the increased hazard of 10-year RFS from the EWAS with Cox model adjusting for immune cell profiles.

**Additional file 5**: The 956 CpGs associated with the decreased hazard of 10-year RFS. The list of the 956 CpGs which were associated with the decreased hazard of 10-year RFS from the EWAS with Cox model adjusting for immune cell profiles.

**Additional file 6**: The results of differentially methylated regions. **no.cpgs**: Number of CpG sites constituting the significant region.

**Additional file 7**: Supplemental tables and figures.

## Data Availability

The datasets generated and analyzed during this current study are available in the gene expression omnibus repository at GSE183920.

## Abbreviations

NMIBC: Non-muscle-invasive bladder cancer
NLR: Neutrophil-to-lymphocyte ratio
mdNLR: Methylation-derived neutrophil-to-lymphocyte ratio
EWAS: Epigenome-wide association study
BCG: Bacillus Calmette–Guérin
CBC: Complete blood count differential
DMRs: Differentially methylated regions
RFS: Recurrence-free survival
OS: Overall survival
HR: Hazard ratio
OR: Odds ratio
CI: Confidence interval
CpG: 5’-Cytosine-phosphate-guanine-3’
DHS: DNase hypersensitive sites
TFBS: Transcription factor binding sites
FDR: False discovery rate
LOLA: Locus overlap analysis
GO: Gene ontology
GSEA: Gene set enrichment analysis
MSigDB: Molecular signature database
