# Supplementary figures and images for: Immune profiles and DNA methylation alterations related with non-muscle-invasive bladder cancer outcomes
Source: Clin Epigenetics. 2022 Jan 21;14:14. doi: 10.1186/s13148-022-01234-6 (PMC8783448; doi:10.1186/s13148-022-01234-6)

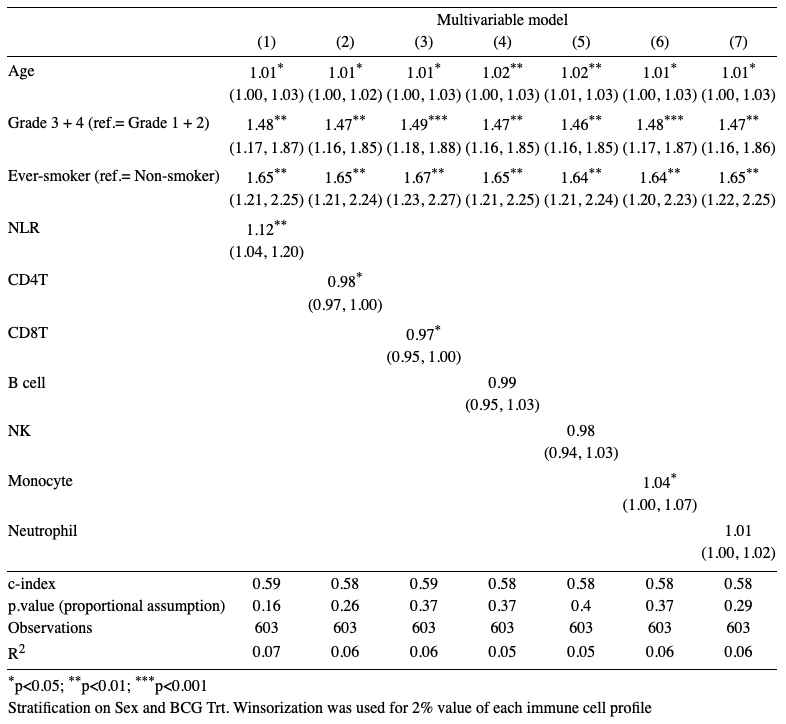

Supplement: Supplementary file 1 — Additional file 1: Cox proportional hazards 10-year recurrence-free survival. Created via using stargazer() function [file 13148_2022_1234_MOESM1_ESM.png]

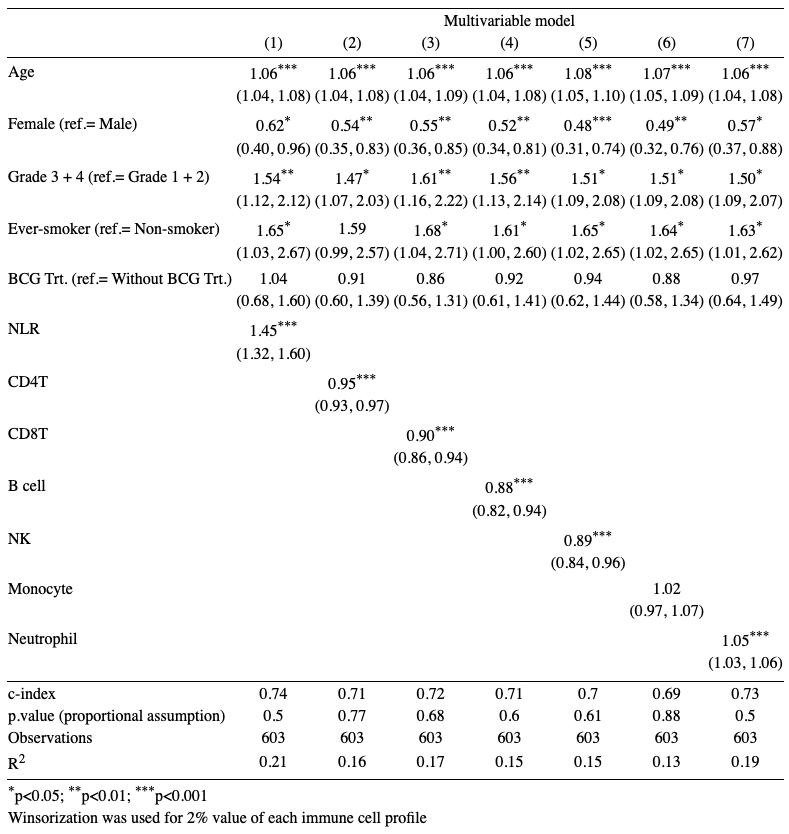

Supplement: Supplementary file 2 — Additional file 2: Cox proportional hazards 10-year overall survival. Created via using stargazer() function [file 13148_2022_1234_MOESM2_ESM.png]
