# Supplementary material for: Immune profiles and DNA methylation alterations related with non-muscle-invasive bladder cancer outcomes
Source: Clin Epigenetics. 2022 Jan 21;14:14. doi: 10.1186/s13148-022-01234-6 (PMC8783448; doi:10.1186/s13148-022-01234-6)
Supplement: Supplementary file 7 — Additional file 7: Supplemental tables and figures. [file 13148_2022_1234_MOESM7_ESM.docx]

Supplemental Figure 1.

Summary of each immune cell profile from DNA methylation deconvolution (603 NMIBC patients). (A) Leukocyte proportions (B) Neutrophil percent (C) Methylation-derived neutrophil-lymphocyte ratio. For Leukocyte proportions (panel A) and NLR (panel B), winsorization was used on the top 2% value, and the red dashed line indicates the 98th percentile of each immune cell profiles. For neutrophil percent, winsorization was used on the bottom 2% value, and the red dashed line indicates the 2th percentile of neutrophil percent.

Supplemental Figure 2.

Kaplan-Meier analysis of 10-year overall survival (OS). 10-year OS curves stratified by (A) age, (B) sex, (C) tumor grade, (D) smoking status, (E) BCG treatment status or (F) mdNLR level. P-values for Log-rank tests are shown.

Supplemental Figure 3A.


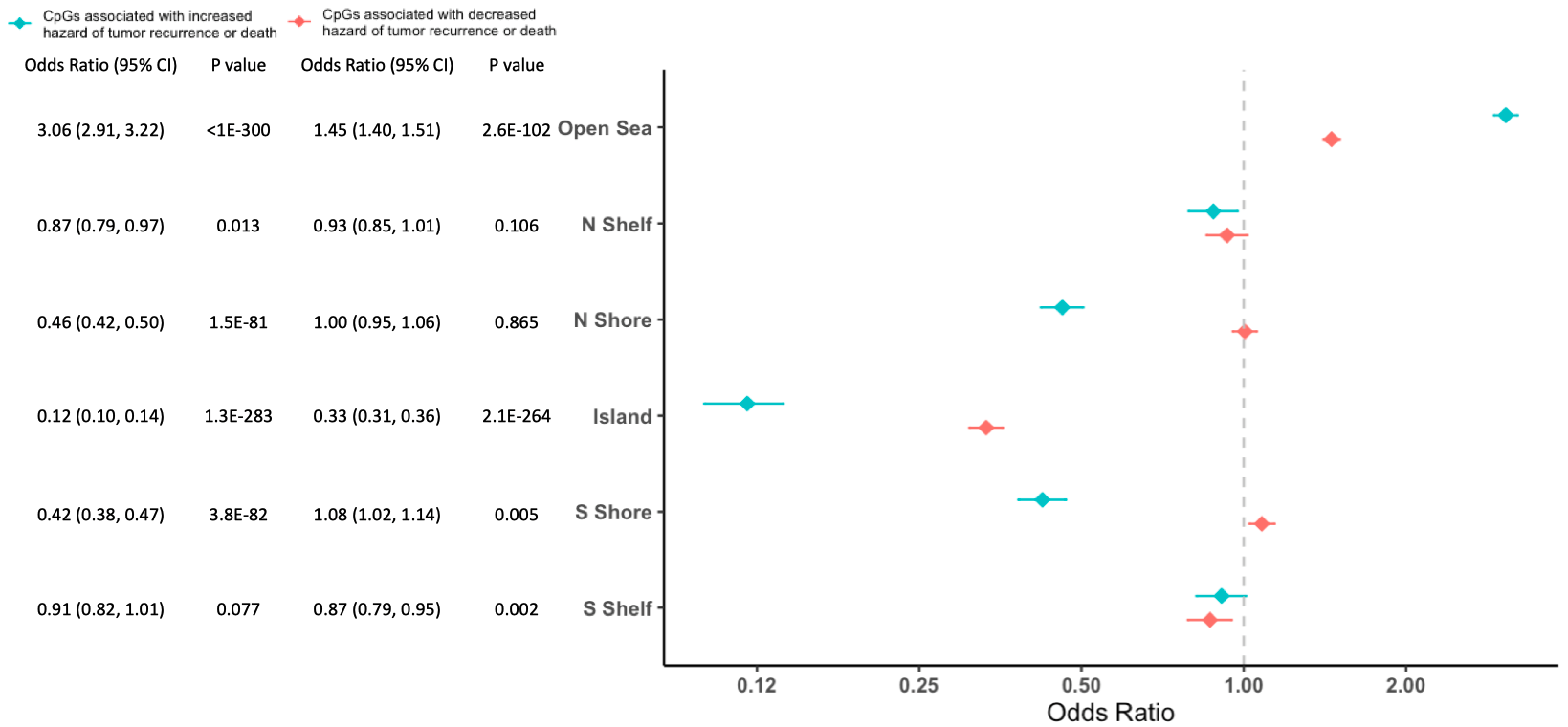


Supplemental Figure 3B.


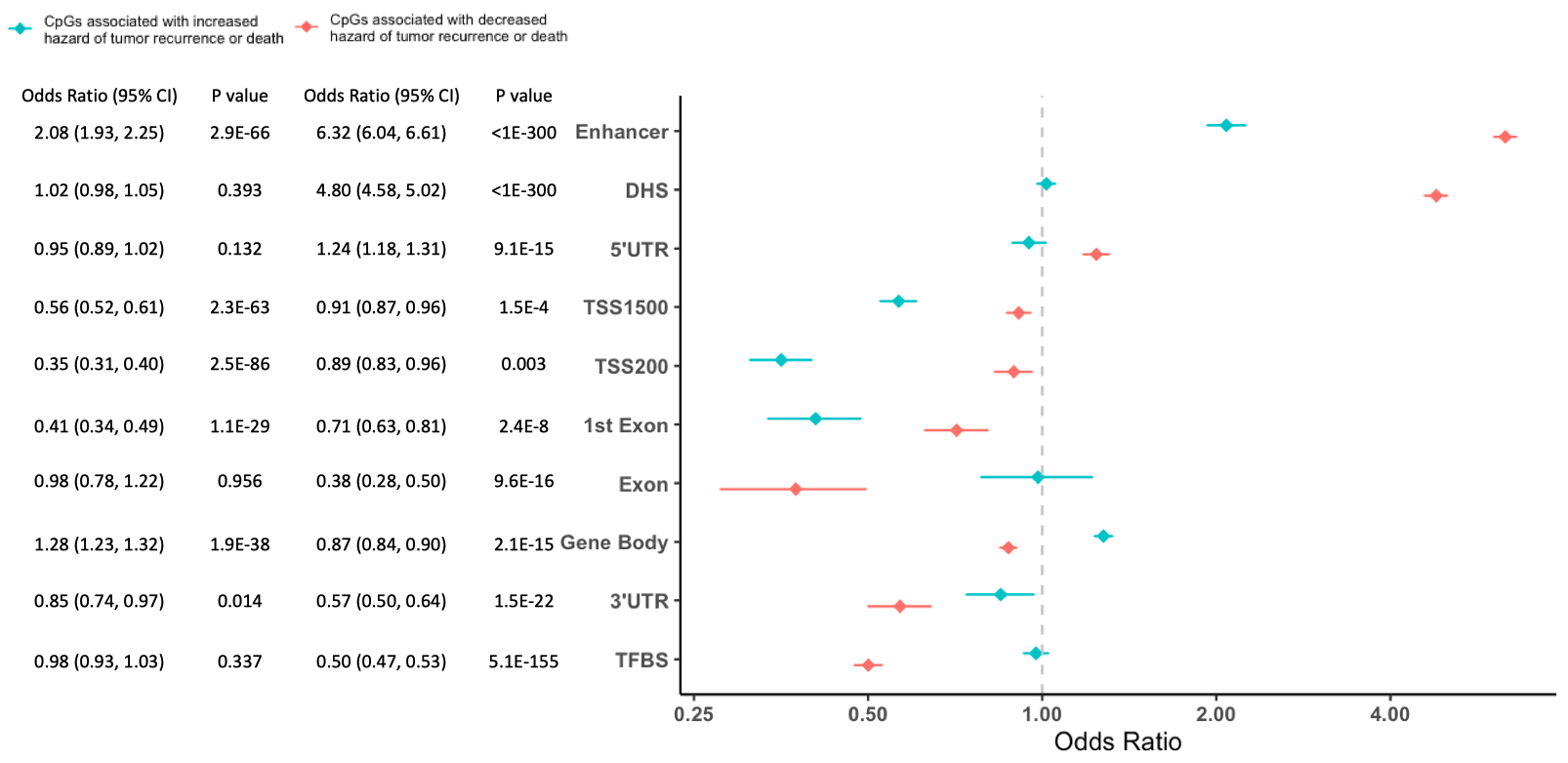


Enrichment analysis of (A) relation to CpG island and (B) genomic context of NMIBC recurrence-free survival associated CpGs. The 27,575 CpGs from EWAS (P-value < 0.005) without adjusting immune cell profiles were tested for enrichment versus all modeled CpGs. The bar represents the 95% confidence interval. Mantel-Haenszel test was used to test RFS associated CpGs enrichment of CpG island-related gnome context. Odds ratio larger than 1 means enrichment, and odds ratio smaller than 1 indicates depletion.

Supplemental Figure 4A.


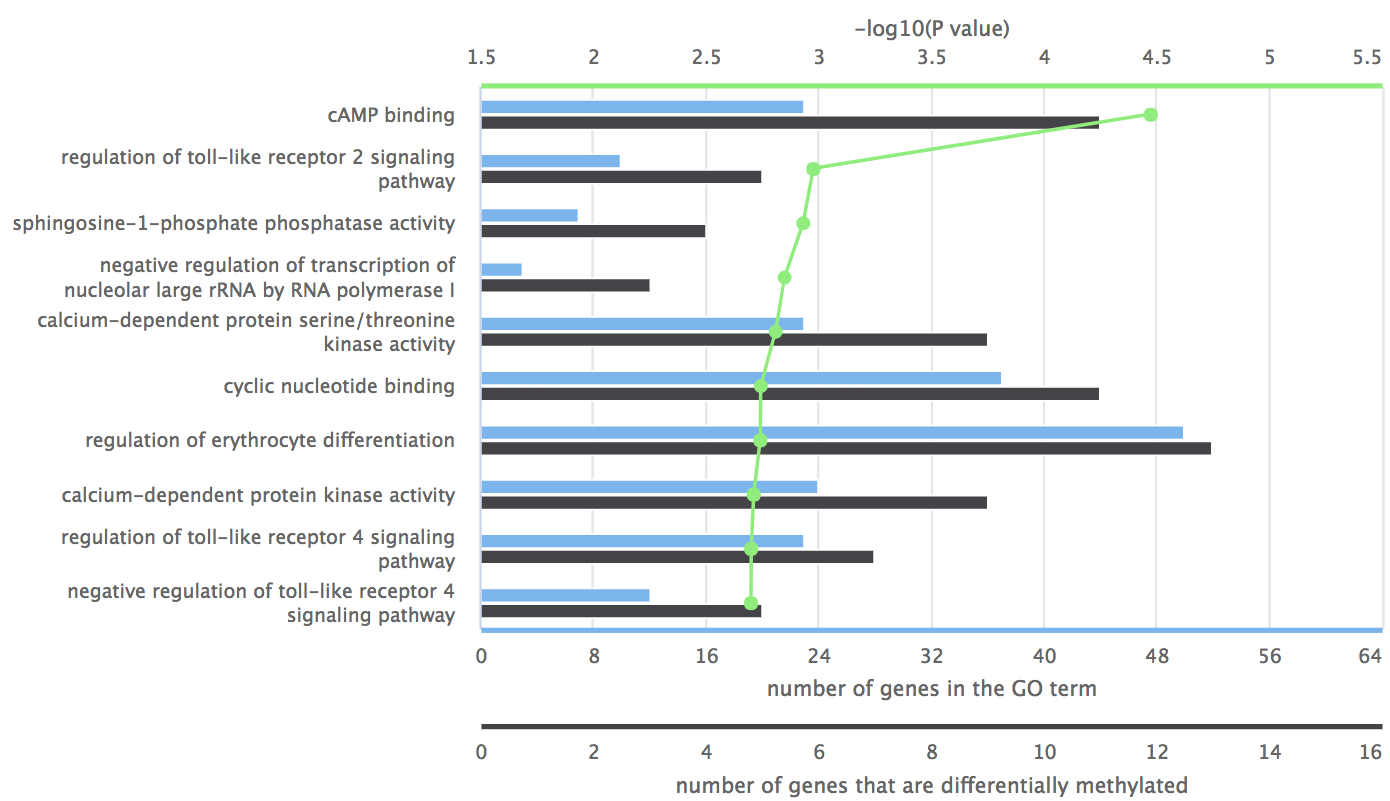


Supplemental Figure 4B.

Supplemental Figure 4C.


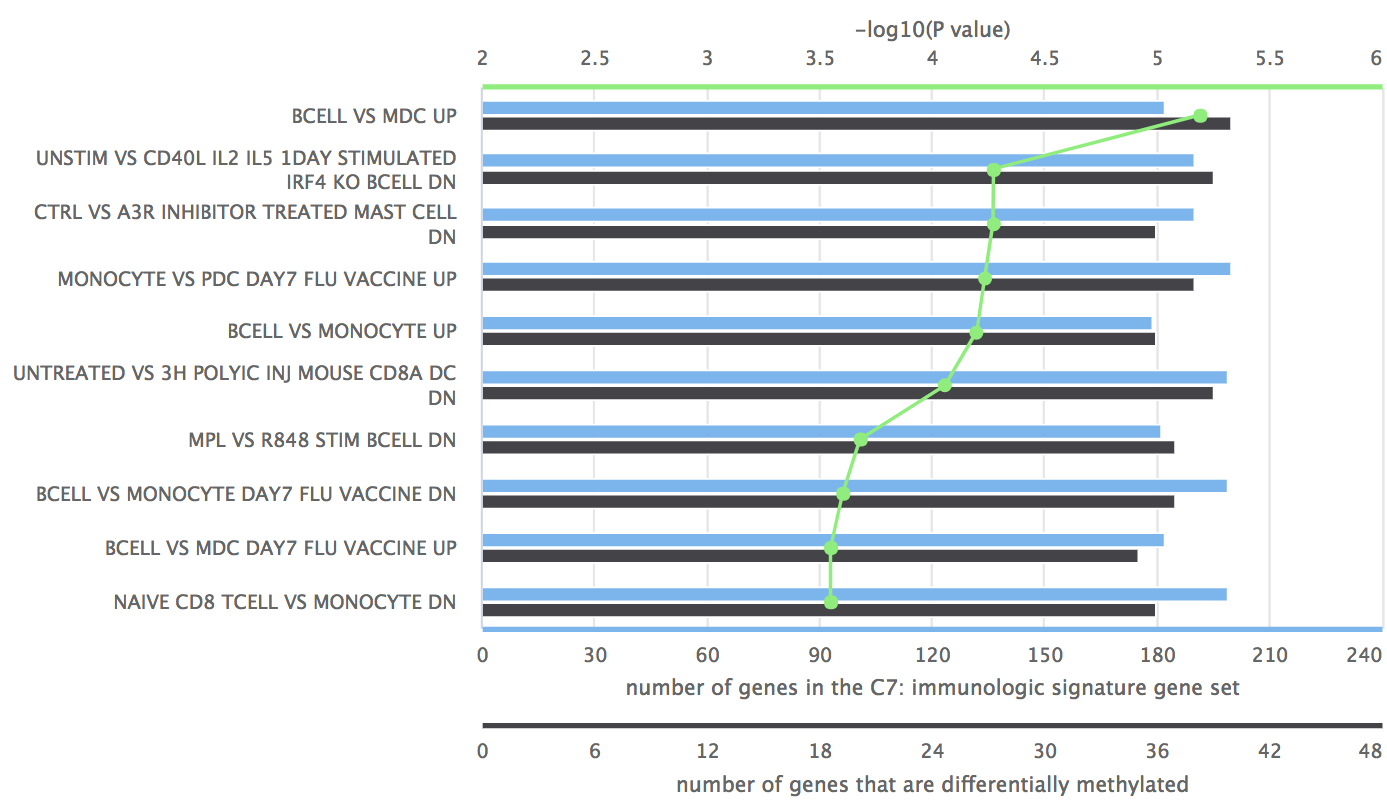


Supplemental Figure 4D.

Gene set enrichment analysis (GSEA). The input was 2,528 RFS associated CpGs from the EWAS which Cox proportional multivariable model with adjusting immune cell profiles were fitted in. (A, C) The top green axis is corresponded to negative log 10 unadjusted p-value, and the further to the right, the more significant; the bottom blue axis is represented to the total number of genes in the gene set; the black axis is corresponded to the number of genes that the hazard-associated CpGs are located in the gene set. (A-B) The results of GSEA for the gene ontology (GO) term. (A) Only the top 10 RFS associated pathways are shown. (B) Gene-concept network is displayed for the top 5 pathways. (C-D) The results of GSEA for the immunologic signature gene set. (C) the top 10 RFS associated pathways. (D) gene-concept network for the top 5 pathways.

Supplemental Figure 5A.


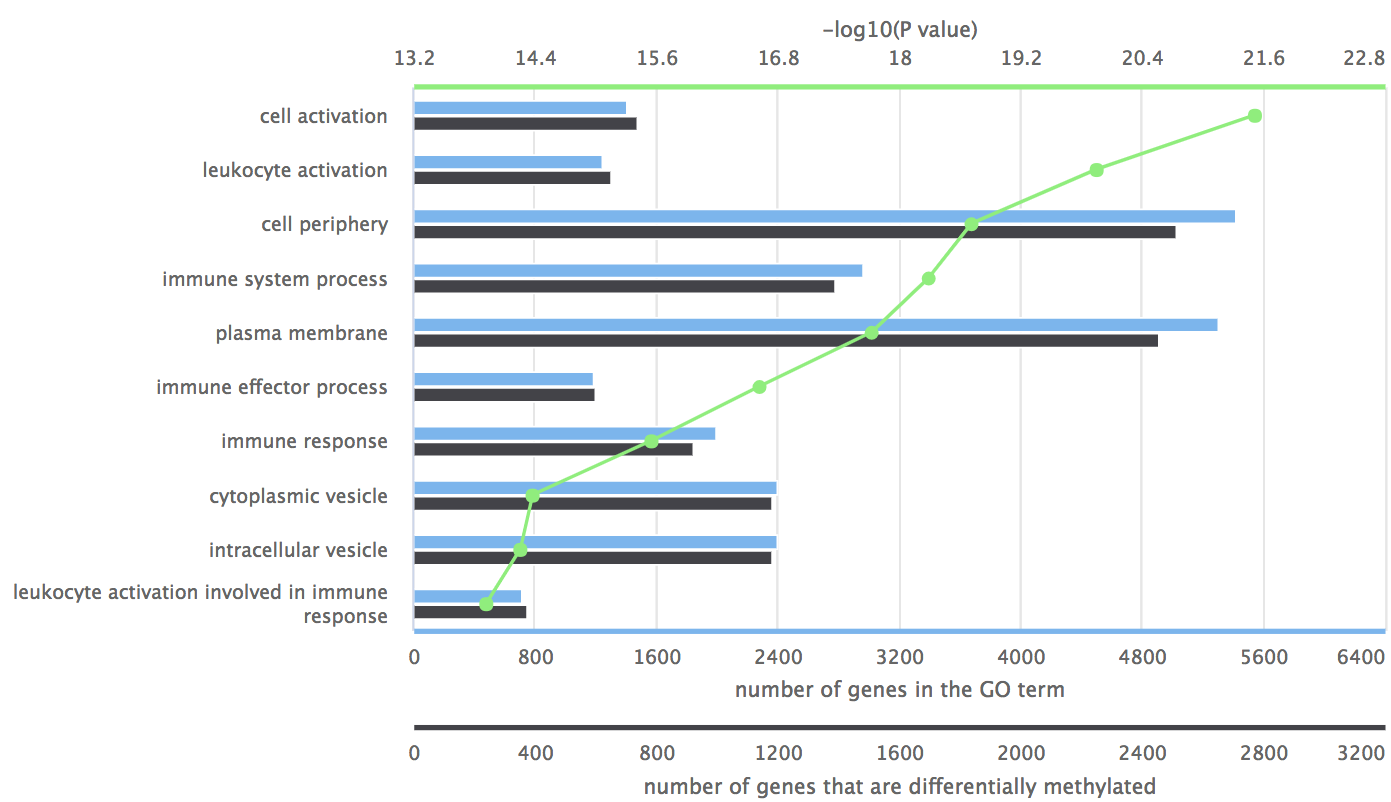


Supplemental Figure 5B.

Supplemental Figure 5C.


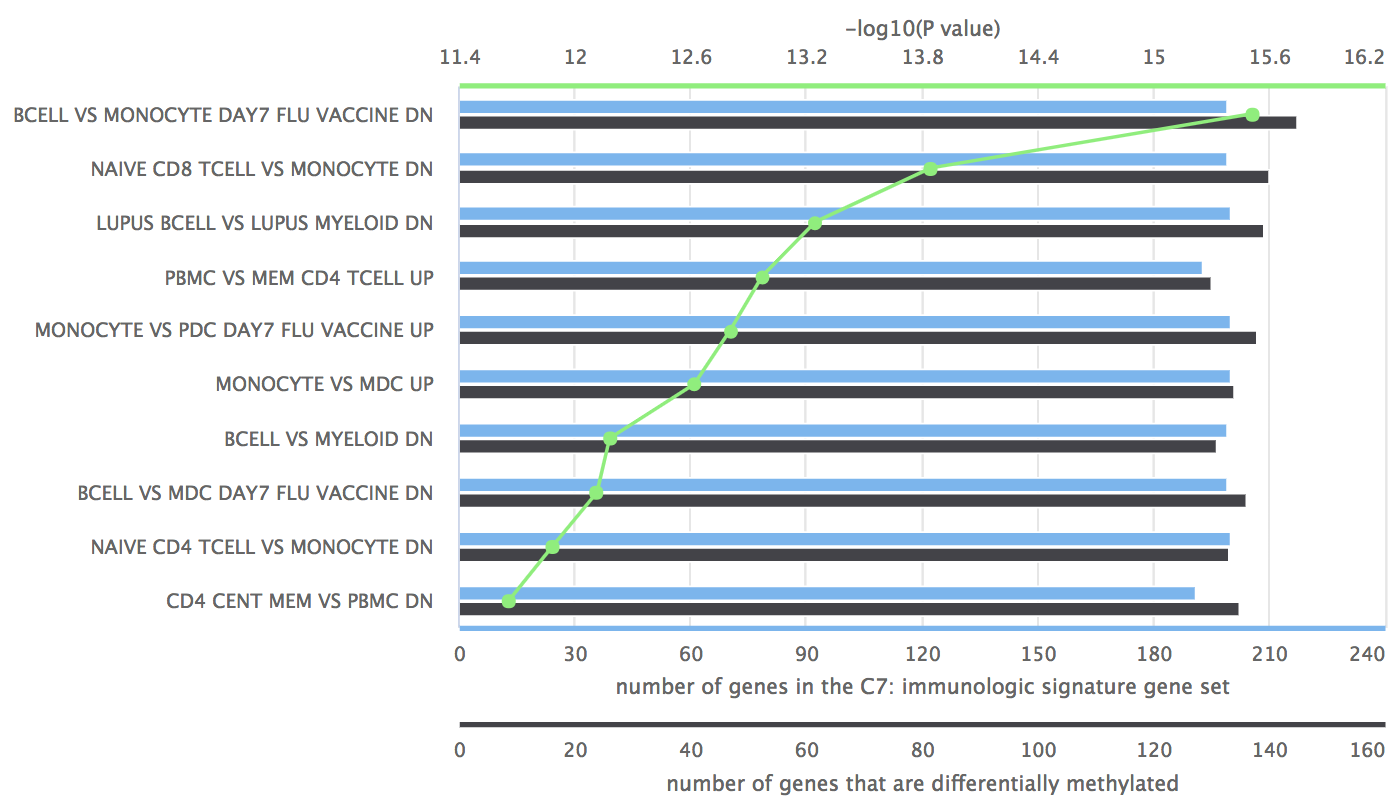


Supplemental Figure 5D.

Gene set enrichment analysis (GSEA). The input was 27,575 RFS associated CpGs from the EWAS which Cox proportional multivariable model without adjusting immune cell profiles were fitted in. (A-B) the results of GSEA for the gene ontology (GO) term. (A) the top 10 RFS associated pathways. (B) gene-concept network for the top 5 pathways. (C-D) the results of GSEA for the immunologic signature gene set. (C) only the top 10 RFS associated pathways are shown. (D) gene-concept network for the top 5 pathways.

Supplemental Figure 6A.


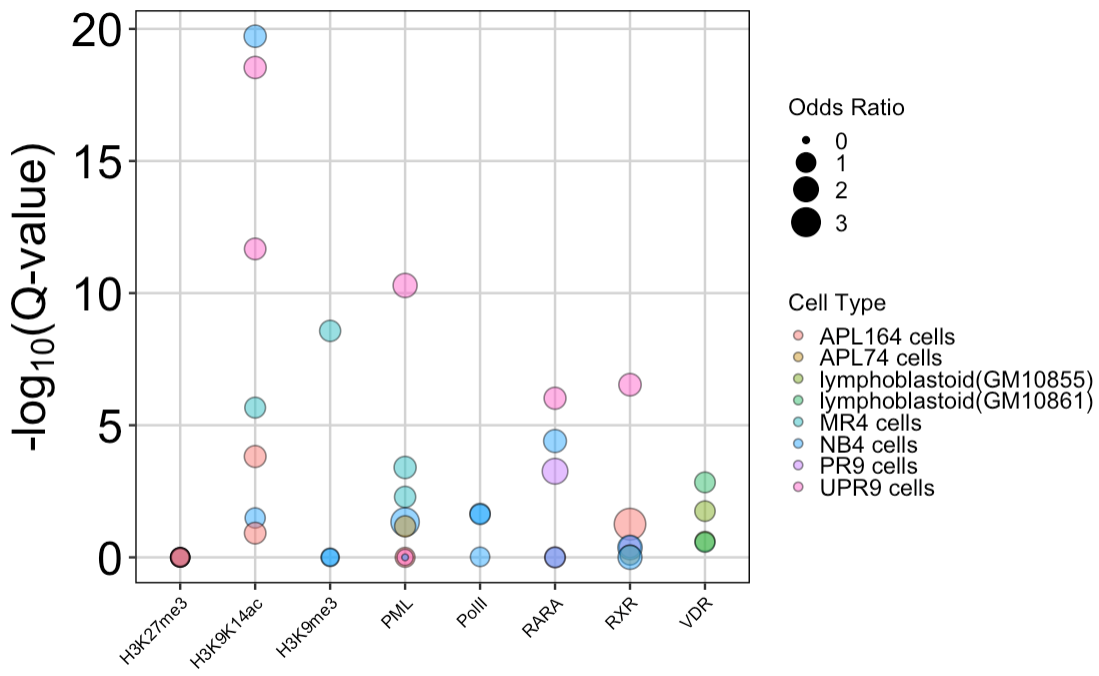


Supplemental Figure 6B.


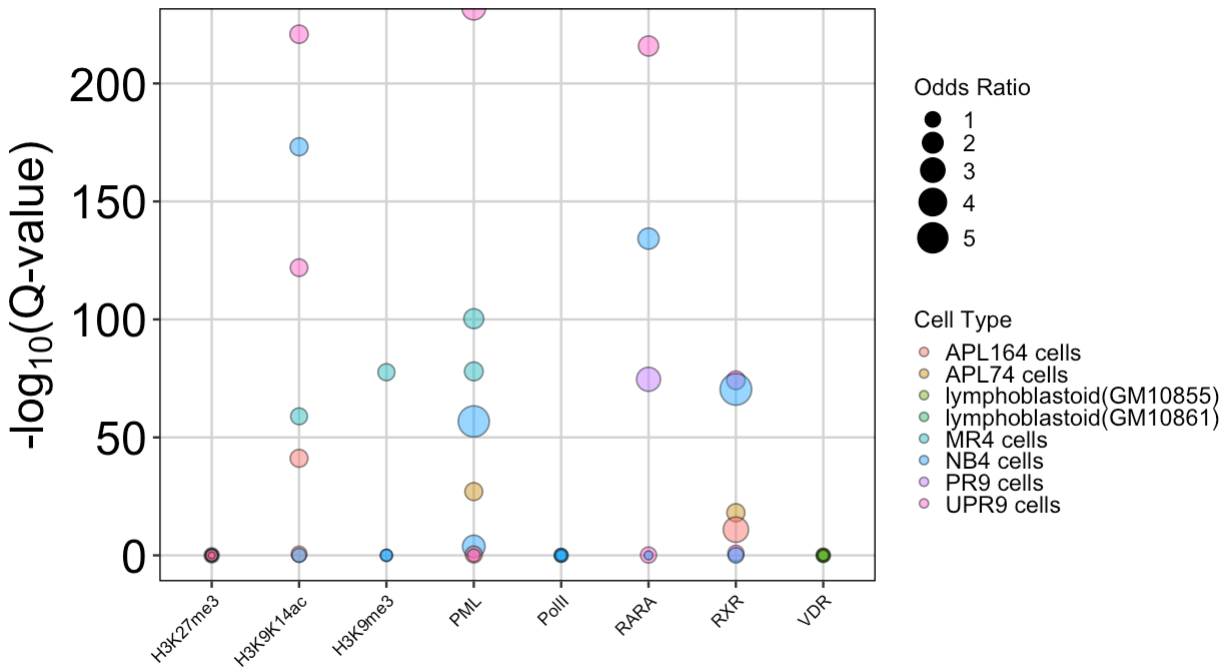


Enrichment of NMIBC recurrence-free survival associated (P-value < 0.005) CpGs from EWAS using locus overlap analysis (LOLA). Cell types were limited to “hematopoiet stem cell”. (A) the input was 2,528 RFS associated CpGs from the EWAS which Cox proportional multivariable model with adjusting immune cell profiles were fitted in. (B) the input was 27,575 RFS associated CpGs from the EWAS which Cox proportional multivariable model without adjusting immune cell profiles were fitted in.

Supplemental Figure 7A.


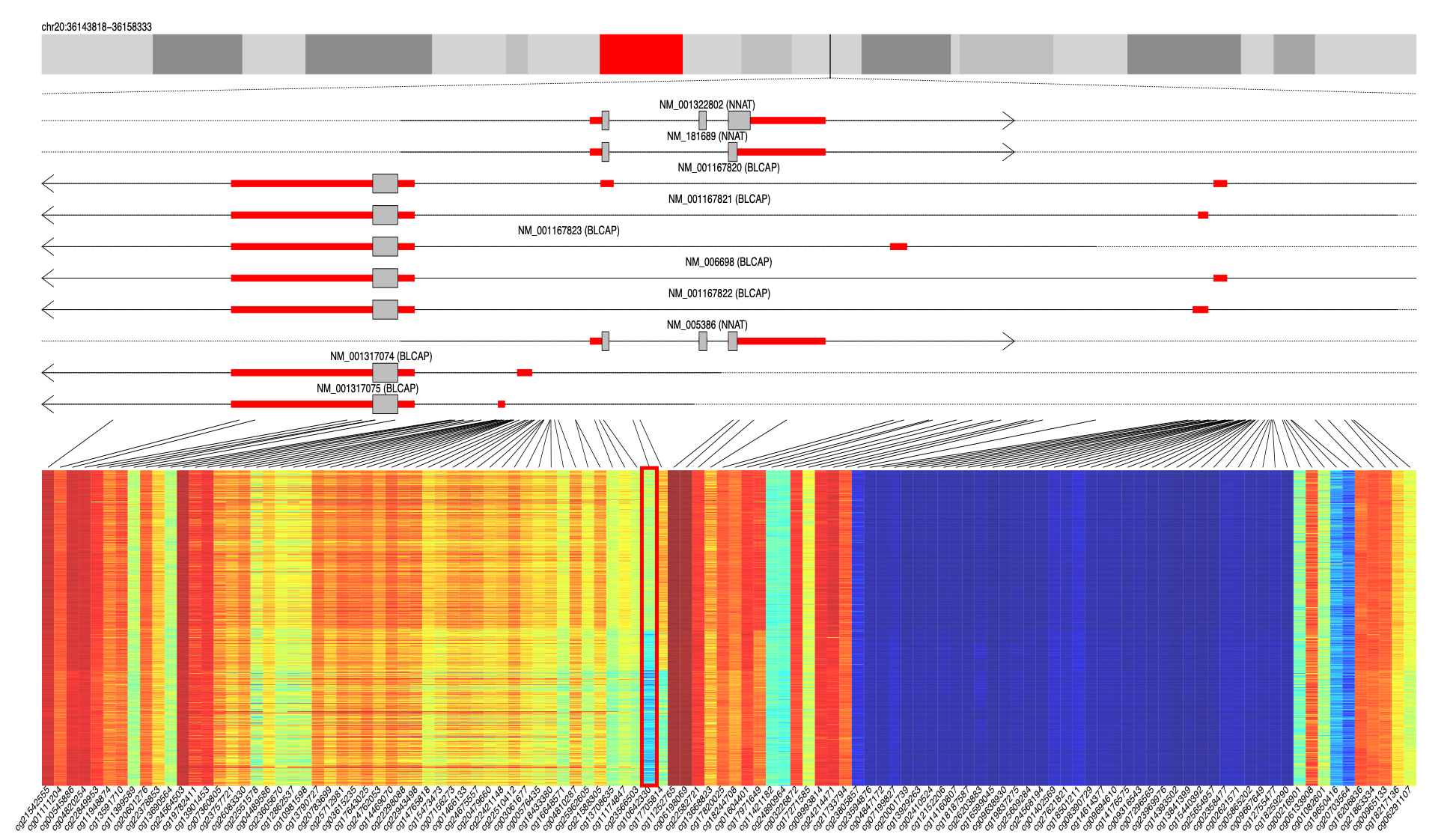


Supplemental Figure 7B.


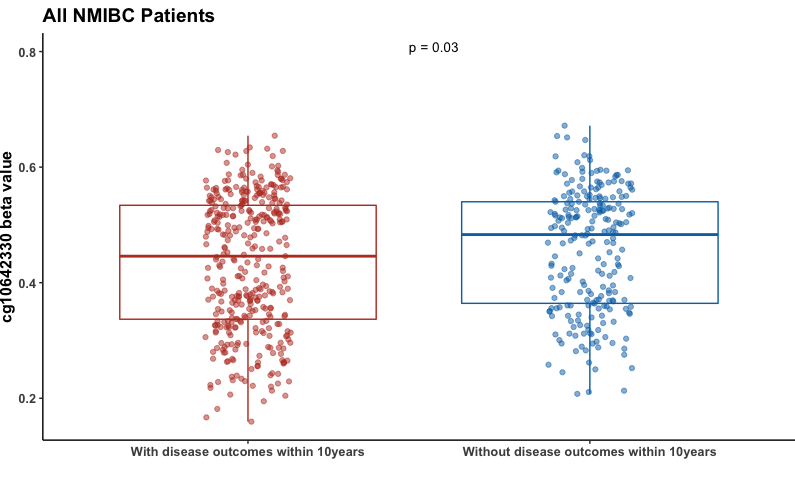


Supplemental Figure 7C.


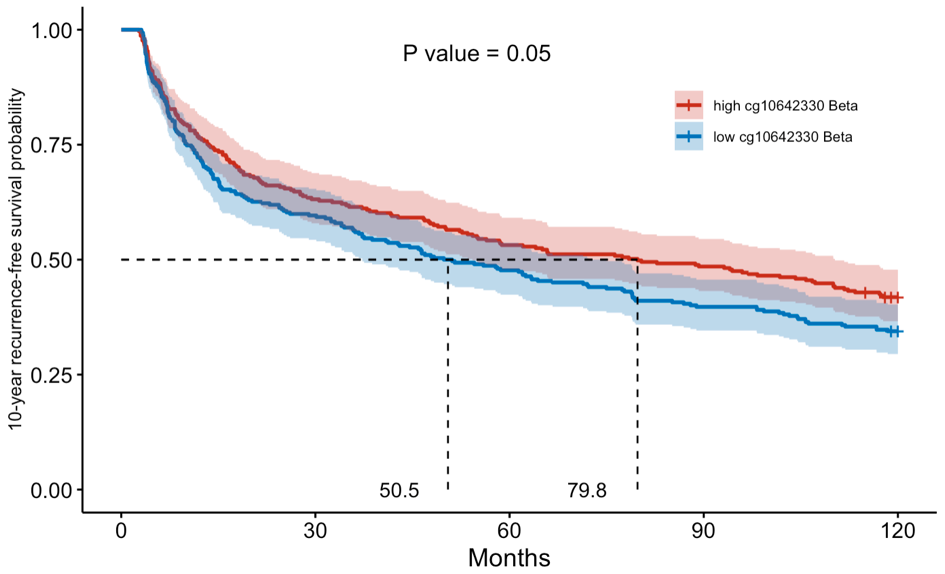


The distribution of methylation levels in the *BLCAP* gene region of each NMIBC patients (A) In the heatmap, each row represents a subject; cg10642330 is labeled in the red box. (B) The distribution of cg10642330 beta values within NMIBC patients. Disease outcomes: death or tumor recurrence. P-values for Wilcox tests is shown. (C) NMIBC patients were grouped according to the median of cg10642330 methylation levels, and the KM plot for recurrence-free survival was shown.
